# The Implications of Extensive Drug-resistant Typhoid Fever: A Case Report

**DOI:** 10.7759/cureus.5032

**Published:** 2019-06-29

**Authors:** Azhara Binte Azhar, Amnah Khalid, Sabeeka Shah

**Affiliations:** 1 Internal Medicine, Shifa College of Medicine, Shifa International Hospital, Islamabad, PAK; 2 Public Health, Shifa College of Medicine, Shifa International Hospital, Islamabad, PAK

**Keywords:** xdr typhoid, enteric fever, pakistan, infectious diseases, s typhi, public health, antibiotic resistance

## Abstract

Salmonella Typhi (S.Typhi) is the causative agent in typhoid fever. In Pakistan, an extensive drug-resistant (XDR) S.Typhi strain has emerged that is resistant to all recommended antibiotics, including third-generation cephalosporins. We report the case of a 29-year-old pregnant woman presenting with high-grade fever, lower abdominal pain, concerns of urinary burning, and increased urinary frequency lasting four days. Blood cultures confirmed XDR S. Typhi. This case highlights three important items: the emergence of the XDR typhoid strain in an unstudied community, the susceptibility of immunocompromised individuals to infectious diseases, and the role health care practitioners can play in controlling its spread regionally and globally.

## Introduction

The causative agent of typhoid fever, Salmonella Typhi (S.Typhi), has developed a resistance to antibiotic treatments, creating multi-drug resistant (MDR) strains. MDR strains are unaffected by traditional first-line antibiotic drugs such as chloramphenicol, ampicillin, and trimethoprim-sulfamethoxazole. MDR strains also have sporadic resistance against second-line drugs, such as fluoroquinolones, especially in parts of Asia and Africa. In Pakistan, an extensive drug resistant (XDR) S. Typhi strain has emerged that is resistant to all recommended antibiotics for typhoid fever, including first-line and second-line drugs as well as third-generation cephalosporins. The Pakistan Health Authorities documented the outbreak of XDR typhoid fever cases from 2016 to 2018 in the province of Sindh, citing 5,274 cases of XDR typhoid of a total of 8,188 typhoid fever cases [1]. The mechanism of transformation of the bacteria from MDR to XDR occurs via the single step of acquiring a plasmid. In Pakistan, typhoid fever remains common in places with poor sanitation and unsafe drinking water, leaving a large segment of the population at risk, especially those with compromised immunity due to the higher chances of complications. The spread of the new XDR strain is alarming and requires immediate action. The XDR strain is sensitive to a handful of drugs such as azithromycin, which is intolerable for some patients. Therefore, the overuse of broad-spectrum antibiotics like meropenem can lead to untreatable strains and severe complications from typhoid, resulting in an epidemic.

## Case presentation

A 29-year-old woman presented to the ED with high-grade fever. She was pregnant, Gravid V, Para II, Aborta II, at 18 + 6 weeks of gestation. She reported a fever (105°F), lower abdominal pain, and increased urinary frequency with burning for four days. Her fever was continuous, accompanied by chills and rigors, with no aggravating or relieving factors. She came to the emergency department (ED) after her symptoms worsened to include vomiting and diarrhea. She reported four episodes of vomiting and was not tolerating anything orally, and she reported eight episodes of diarrhea in one day. The diarrhea was watery, non-bloody, and accompanied by lower abdominal pain. She had not traveled recently, nor had she been in contact with sick people.

On examination, the patient was well-orientated but lethargic and in obvious distress. Her temperature was 101°F; her blood pressure was 100/60 mmHg; her pulse was 75 beats per minute, and her Glasgow coma scale (GCS) score was 15/15. On chest examination, we detected no added heart sounds or murmurs, no wheezes or crackles, and her breath sounds were clear and equal, bilaterally. Her vaginal speculum examination was deferred to prevent the introduction of bacteria into the genital tract.

The results of her rubella and toxoplasmosis immunoglobulin G titers were insignificant. The results of screens for syphilis, hepatitis A, B, and C, dengue, and rapid malaria test were negative. Her C-reactive protein level was elevated at 103.3 mg/L, causing suspicion for cystitis/pyelonephritis given her urinary concerns. Blood and urine samples were drawn for culture and sensitivity. The patient was empirically started on intravenous (IV) ceftriaxone. However, her fever did not subside, and her condition worsened with continued rigors, chills, and diarrhea. The patient’s laboratory results during hospitalization are presented in Table 1.

**Table 1:** Laboratory analysis during hospitalization WBCs, white blood cells; BUN, blood urea nitrogen; AST, aspartate transaminase; ALT, alanine transaminase; CRP, c-reactive protein

Analyte	Day 1	Day 2	Day 5	Day 7
Hemoglobin (g/dL)	11.6	9.1	8.2	8.7
WBC (µL)	6400	4650	3180	4890
Platelets (x10^3^/µL)	166	123	113	112
Sodium (mEq/L)	131	138		
Potassium (mEq/L)	3.6	3.7		
Chloride (mEq/L)	98	106		
BUN (mg/dL)	3			
Urea (mg/dL)	6.42			
Bicarbonate (mEq/L)	21	18		
Creatinine (mg/dL)	0.5			
AST (U/L)	106		394	175
ALT (U/L)	60		189	112
CRP (mg/L)	103.39	82		
Random glucose (mg/dL)	87	95		

The results of the patient’s routine urine examination and urine culture were unremarkable. Blood Gram stain showed the presence of Gram-negative rods. After consultation with an infectious disease specialist, the patient was started on meropenem (2 g IV immediately, 1 g every eight hours) before receiving the results of the blood culture report. The blood culture and sensitivity report was positive for S. Typhi (extended-spectrum beta-lactamase; Table 2).

**Table 2:** Blood culture and sensitivity report ESBL, extended spectrum beta-lactamase; R, resistant; S, sensitive

Antibiotics	Salmonella Typhi (ESBL)
Ampicillin	R
Azithromycin	S
Cefixime	R
Ceftriaxone	R
Chloramphenicol	R
Ciprofloxacin	R
Co-Trimoxazole	R
Imipenem	S
Meropenem	S

On the sixth day of hospitalization, her condition improved. Her body temperature was 99°F, and the vomiting and diarrhea subsided. She was prescribed meropenem (1 g) every eight hours for a total of seven days. She is currently being observed as an outpatient.

## Discussion

The outbreak of XDR S. Typhi cases in Pakistan has become an alarming public health concern. Since 2016, 5,274 cases have been reported in Pakistan, the majority of which are in districts of Sindh, including Karachi and Hyderabad [1].

The management of MDR S. Typhi (i.e., resistant to first-line drugs) has been dependent on azithromycin and cephalosporins. The recent emergence of XDR S. Typhi strains (i.e., resistant to both first-line and second-line treatments) has made effective management difficult [2]. This is particularly complicated when dealing with immunocompromised patient populations such as children, elderly, and pregnant women. Compounding the difficulty of managing these cases in developing countries is the lack of resources and the cost of new medications [3].

Potential treatment options available for XDR S. Typhi include azithromycin, carbapenems, tigecycline, piperacillin-tazobactam, ceftazidime-avibactam, fosfomycin, and colistin. The efficacy of these drugs for XDR S. Typhi has not yet been proven. More evidence-based research is required to determine an effective treatment regimen for these cases to avoid monotherapy [3]. Our patient was treated with meropenem. While this monotherapy regimen proved to be effective in this case (with the patient eventually being managed on an outpatient basis), its use may promote further drug resistance. The lack of an effective oral therapy regimen for XDR S. Typhi can lead to a shift in the treatment setting from mainly being an outpatient approach to becoming predominantly inpatient treatment, further increasing the burden in hospitals [4].

Another potential cause for antibiotic resistance in Pakistan is the lack of fully equipped and standardized laboratories in rural settings. Such laboratories lack resources to process blood cultures and accurately report S. Typhi strain sensitivity and resistance, leading to ineffective treatment in addition to drug resistance. Public health initiatives and funding are required to ensure that proper diagnostic technology is readily accessible in rural areas with a heavy disease burden.

As S. Typhi is spread through fecal-oral contamination, preventive measures to reduce spread will decrease the incidence of the disease. Public health involvement is crucial to improve water quality, create proper sanitation facilities, and increase public awareness. The water and sanitation problem in the country is a major setback for the treatment of infectious diseases like typhoid, which continue to remain endemic. There needs to be a shift in thinking to promote solutions and more sustainable, long-term goals. Effective and sanitary sewage disposal needs to become a priority for municipalities. Safe water filtration plants need to be well-maintained and accessible to the general population.

Implementation of awareness programs through the ministry of health in basic health units, rural health centers, and secondary and tertiary care hospitals for the general patient population are much needed. Education should focus on the mechanism of transmission of typhoid, basic hygiene, hand washing, exposure to contacts, and incubation periods; these could be in the form of posters, within network commercials, and counseling on prevention measures from a primary health care contact.

The importance of vaccination should be stressed, especially if herd immunity is to develop, which helps curtail the spread of disease. The vaccination is easy to administer and should be considered a priority within areas where the disease is endemic. The conjugated Vi polysaccharide vaccine, Typbar-TCV, was approved by the World Health Organization (WHO) in 2018 for use in infants older than six months [5]. This vaccine provides prolonged immunogenicity. The recommended schedule is a single dose given intramuscularly in children aged six to 23 months and a catch-up vaccination in children aged two to 15 years [3]. The prevalence of this vaccination has been limited in Pakistan due to cultural practices and a lack of public knowledge and understanding [5]. Further initiatives to increase vaccination compliance and education are critical to controlling the spread of disease.

Lastly, pharmaceutical regulatory bodies in Pakistan need to further scrutinize private pharmacy vendors selling antibiotics without prescriptions. Along with overuse, the over-prescription of antibiotics by physicians could also be a rising problem given the development of bacterial drug resistance [6]. There needs to be a focus on education specific to the rising problem of antibiotic resistance and inefficient prescription of antibiotics for newly trained doctors and those entering formal medical education.

## Conclusions

XDR typhoid is an emerging concern at a global level and needs to be addressed through simple but effective public health measures. There continues to be extensive research on the mechanism of resistance of the XDR strain and new antibiotic susceptibility, which will be helpful in the long term. However, the prevention and control of the impact of the spread of typhoid is critical and may be achieved through improving water and sanitation, hygiene awareness and hand washing, successful vaccination programs, and availability of standardized laboratory facilities, and by reducing the inappropriate use and prescribing practices for antibiotics.
